# Correspondence of ‘Evaluation of large language models in breast cancer clinical scenarios: a comparative analysis based on ChatGPT-3.5, ChatGPT-4.0, and Claude2’

**DOI:** 10.1097/JS9.0000000000001616

**Published:** 2024-05-15

**Authors:** Fangchu Lo, Kahei Au, Wah Yang

**Affiliations:** a School of Medicine, Jinan University; b Department of Metabolic and Bariatric Surgery, The First Affiliated Hospital of Jinan University, Guangzhou, Guangdong Province, People’s Republic of China

*To the Editor,*

Artificial intelligence (AI) is advancing by leaps and bounds. Lots kinds of new and rapidly changing technologies are on the rise. The most widely discussed is large language models (LLMs). LLMs have garnered significant attention in both AI and medical fields. We are concerned about its foresight use, accuracy, safety, and social acceptance in treatment. We follow the topic ‘Evaluation of large language models in breast cancer clinical scenarios: a comparative analysis based on ChatGPT-3.5, ChatGPT-4.0, and Claude2’ in the *International Journal of Surgery*. The paper study compared the performances of three major LLMs in the clinical context of breast cancer (ChatGPT-3.5, ChatGPT-4.0, and Claude2)^1^. We strongly extend our commendations for their outstanding contributions. However, we believe that several prospective areas exist for enhancement. Therefore, we sought to initiate a discussion regarding the issues of using LLMs in the surgical field.

The LLMs were used to generate feedback for various queries related to five pivotal domains for clinical scenarios of breast cancer in this study. For each scenario, a panel of five breast cancer specialists evaluated the feedback from LLMs. They assessed feedback concerning LLMs in terms of their quality, relevance, and applicability. The method and research object chosen by the author are very prospective and breakthrough, and the results are also significant. However, the relatively insufficient number of experts responsible for the assessment may lead to a decrease in the credibility and accuracy of the assessment.

Consequently, we propose increasing the number of specialists responsible for evaluating feedback. Moreover, the GPT-4.0 Turbo is the latest generation model. It’s more capable and has updated knowledge. GPT-4.0 Turbo can also be considered in the research instead of GPT-3.5, as GPT-3.5 is a bit behind and outdated. GPT-3.5’s performance, data updates, and prospects in the medical field are significantly behind GPT-4.0. Further research can consider GPT-4.0, GPT-4.0 Turbo, and Claude2, which may be more forward-looking, applicable, and closer to the current new progress. Furthermore, explainability is one of the most discussed topics regarding the application of AI in the surgical field, and it needs to be mentioned more. Also, ethical issues, data security, bias, and generalizability are significant challenges when using LLMs in the surgical field. These issues require further exploration in the use and research of LLMs. Solving the above problems can more effectively implement and promote the application of LLMs. We appreciate the result that was conducted by the author, and we strongly agree that GPT-4.0 has consistently shown excellent performance in studies, indicating its potential and prospects in clinical applications.

LLMs can respond to free-text queries without requiring specialized training for the task in question, raising both excitement and concern about their use in healthcare settings^2^. LLMs can be used as a supporting tool in surgical diagnosis and treatment^3^. It can used for tasks such as assisting diagnosis, giving predictions of diseases, and analyzing medical cases. LLMs can serve as an auxiliary tool to help healthcare professionals. Often, an LLM can be crucial but requires human oversight and ethics. LLMs remain inferior to clinicians^4^. It is by no means a substitute for professional medical advice. When used in the medical field, professional supervision is essential and noticeable. In performing a medical act, the one operating the GPT must be able to interpret and process the results generated, make informed decisions based on clinical judgment, and check the authenticity of the information. In this new era of digital life and AI, the potential of LLMs for medical applications in diagnosis and treatment is huge. Still, research, training, consensus, and guidelines are needed to promote its application. At the same time, researchers need to deepen society’s trust in LLMs to maximize their effectiveness and acceptance. The potential of GPT is huge, with accuracy and usability already proven. In some cases, GPT may be of benefit to healthcare workers. GPT can provide relevant literature and information to assist those who need to find the latest research or treatment options for a particular disease. It can provide information to provide more convenient support. Moreover, as the world becomes increasingly connected, GPT has the potential to revolutionize travel medicine to avoid health risks^5^. More research is needed to demonstrate the effectiveness, accuracy, and reliability in the surgical field.

## Ethical approval

Not applicable.

## Consent

Not applicable.

## Sources of funding

Not applicable.

## Author contribution

F.L. and W.Y.: conceptualization, methodology, and formal analysis; W.Y.: resources, supervision, and project administration; F.L.: writing – original draft preparation; K.A. and W.Y.: writing – review and editing.

## Conflicts of interest disclosure

The authors declared no conflicts of interest.

## Research registration unique identifying number (UIN)

Not applicable.

## Guarantor

Wah Yang.

## Data availability statement

Not applicable.

## Provenance and peer review

Not applicable.
